# Chromovitrectomy: current evidence, emerging technologies, and practical applications in vitreoretinal surgery

**DOI:** 10.1007/s00417-026-07307-z

**Published:** 2026-06-05

**Authors:** Federico Giannuzzi, Lorenzo Hu, Mattia Cusato, Matteo Mario Carlà, Emanuele Crincoli, Federico Maragno, Valentina Cestrone, Umberto De Vico, Stanislao Rizzo

**Affiliations:** 1 Ophthalmology Unit, “Fondazione Policlinico Universitario A. Gemelli, IRCCS”, Largo A. Gemelli, 8, Rome, Italy; 2 Università Cattolica del Sacro Cuore, Rome, Italy; 3 Department of Ophthalmology, Hopital Fondation Adolphe De Rothschild, Paris, France; 4 Consiglio Nazionale delle Ricerche, Istituto di Neuroscienze, Pisa, Italy

**Keywords:** Chromovitrectomy, Vital dyes, Brilliant blue G, Trypan blue, Internal limiting membrane, Digital visualization, Phototoxicity

## Abstract

Chromovitrectomy has revolutionized vitreoretinal surgery by enabling the visualization of nearly transparent intraocular structures. This review evaluates the clinical utility of key vital dyes, such as brilliant blue G, trypan blue, indocyanine green, and triamcinolone acetonide, focusing on their physicochemical properties, staining affinities, and safety profiles. Recent advancements in dye formulation, including high-density and multi-component preparations, have improved tissue selectivity and surgical handling. These pharmacological innovations are now complemented by digital visualization technologies, such as 3D heads-up displays and customizable spectral filtering, which enhance contrast perception and ergonomics. We further discuss specialized surgical strategies for identifying occult retinal breaks and sequential staining techniques, while addressing critical concerns regarding phototoxicity and macular protection. As the field moves toward integrating intraoperative OCT and artificial intelligence, chromovitrectomy remains a dynamic cornerstone of posterior segment surgery. Understanding the synergy between novel biocompatible agents and digital platforms is essential for optimizing surgical precision and patient outcomes.

**Supplementary Information:**

The online version contains supplementary material available at https://doi.org/10.1007/s00417-026-07307-z.

## Abstract (fr)

**Key messages:**

***What is known*:**

- Vital dyes (BBG, trypan blue, ICG, triamcinolone) are indispensable for visualizing transparent vitreoretinal structures, but each carries a concentration- and light-dependent toxicity profile.

- BBG and BBG/trypan blue combinations have largely replaced ICG for ILM and ERM peeling due to superior selectivity and safety.

***What is new*:**

- Newer formulations, heavy dual dyes (DDG/diglycerol) and lutein-based preparations, allow lower dye concentrations while adding intrinsic photoprotection.

- 3D heads-up displays with spectral filters enable reduced endoillumination, offering a synergistic strategy with safer dyes to limit phototoxicity.

- Specialized techniques (trypan blue–assisted break identification, sequential under-air/PFCL staining, en-face OCT–guided dye-free peeling) extend chromovitrectomy to complex retinal detachment and personalized macular surgery.

## Introduction

The term “chromovitrectomy” was introduced by Rodrigues et al. in 2005 to describe the application of vital dyes during vitreoretinal surgery to enhance visualization of transparent or semi-transparent structures [1]. This technique has become indispensable in modern pars plana vitrectomy (PPV), enabling surgeons to identify and manipulate the vitreous, internal limiting membrane (ILM), epiretinal membranes (ERM), and proliferative tissues with unprecedented precision.

The evolution from empirical dye application to evidence-based chromovitrectomy reflects advances in understanding dye-tissue interactions and recognition of potential toxicity mechanisms [2]. Contemporary chromovitrectomy encompasses not only dye selection but also consideration of application techniques, exposure duration, illumination parameters, and integration with digital visualization technologies.

This review provides a comprehensive update on chromovitrectomy practice, incorporating recent evidence on dye safety and efficacy, emerging formulations, digital enhancement technologies, and specialized surgical techniques. The goal is to provide vitreoretinal surgeons with practical, evidence-based guidance for optimizing outcomes while minimizing iatrogenic risk.

## Vitreoretinal anatomy

### The vitreous and vitreoretinal interface

Understanding the structural targets of chromovitrectomy requires appreciation of the vitreoretinal interface anatomy. The vitreous body comprises approximately 98% water with a sparse network of collagen fibrils and hyaluronic acid molecules that create its gel-like properties [3, 4].

Type II collagen is the most represented in the vitreous collagen (80%), but the fibrils are heterotypic, so they are also composed by type IX collagen (20%) and type V/XI collagen in negligible amounts [5].

Available evidence indicates that the overall collagen content of the vitreous remains stable during development, shows a modest increase during adolescence, and then stays relatively unchanged throughout adulthood and senescence. While it has been proposed that vitreal collagen production declines or ceases with aging, findings in adult bovine and human eyes support the persistence of collagen turnover into adulthood, with ongoing synthesis even in later life [6, 7]. In the absence of continued collagen synthesis in advanced age, progressive structural degradation would be expected, leading to compromised vitreous biomechanics. Conversely, sustained collagen production would help preserve the mechanical properties of the vitreous, provided that the architecture of the collagen network remains intact.

Hyaluronic acid (HA) is a hydrophilic glycosaminoglycan (GAG) that helps maintain the expansion of the vitreous body, thereby contributing to its resistance to compressive forces. Although the vitreous also contains additional proteins and GAGs, its mechanical behavior and structural integrity are largely determined by the levels and interactions of collagen and HA [8, 9].

The posterior vitreous cortex, approximately 100–300 μm thick, contains the highest collagen concentration and forms the primary attachment to the ILM [10].

Vitreoschisis, splitting within the posterior vitreous cortex, occurs in up to 57% of eyes and can leave residual cortical vitreous adherent to the retinal surface even after apparent posterior vitreous detachment [11]. These vitreous cortex remnants are invisible under standard illumination but become apparent with triamcinolone acetonide (TA) staining, highlighting their importance in macular hole and proliferative vitreoretinopathy pathology [12].

### The internal limiting membrane

The ILM is the basement membrane of Müller cells, forming the innermost boundary of the neurosensory retina. Its thickness varies in the different retinal zones; it is thinnest at the vitreous base and thickens towards the posterior pole, thinning again in the fovea and at the optic nerve [13]. ILM thickness is 0.5–2.0 μm at the fovea and 2.0–3.0 μm in the perifoveal region. The thickness of the ILM showed greater variability at the posterior pole, likely because the membrane becomes more undulating as it adapts to the irregular contour of the Müller cells along its retinal surface. In both the equatorial region and the posterior pole, the ILM was significantly thinner in donors over 60 years of age compared with those younger than 60 [14].

It is a 3-layered structure composed of a lamina rara externa, which comes into contact with the vitreous cortex, a middle layer represented by the lamina densa, and an inner layer, represented by the lamina lucida, adjacent to the endfeet of Müller cells [14]. The ILM comprises predominantly type IV collagen, laminin, fibronectin, and proteoglycans, a composition that determines its staining affinity for specific dyes [15].

Many of these molecules facilitate adhesion between the ILM and the retina, as well as attachment of the cortical vitreous to the ILM [16].

The basement membrane layer of the ILM is thin at the fovea, the optic nerve head, and along the major vascular arcades, where vitreous fibers are firmly and directly attached to the retina. At the foveal center, the Müller cell layer becomes thicker and forms a cone-shaped structure that links the ILM to the external limiting membrane, known as the Müller cap [17].

The differential binding of vital dyes to ILM versus ERM reflects their distinct proteomic compositions. While both contain type IV collagen and laminin, ERMs are characterized by upregulation of type II collagen, cellular elements including glial cells, retinal pigment epithelium cells, and myofibroblasts, and extracellular matrix proteins including fibrillin-1 and tenascin [18]. This compositional difference enables selective staining strategies.

### Epiretinal membranes and proliferative tissues

Epiretinal membranes represent fibrocellular proliferations at the vitreoretinal interface.

Early morphological analyses of surgically removed ERM specimens were limited by the phenotypic transdifferentiation of proliferating epiretinal cells [19, 20]. More recent immunohistochemical studies have demonstrated the participation of multiple cell types in ERM development, including glial cells (Müller cells, fibrous astrocytes, and microglia), fibroblasts, myofibroblasts, hyalocytes, retinal pigment epithelial cells, and macrophages [21, 22].

Although numerous investigations have evaluated a wide range of molecular markers, the precise origin of these cells remains controversial, as findings are frequently inconclusive and sometimes contradictory. Regardless of their source, the cells present in ERMs acquire a myofibroblast-like phenotype, as demonstrated by the expression of α-smooth muscle actin (α-SMA) and their capacity to produce and deposit new extracellular matrix [21, 23–27].

Also scanning electron microscopy (SEM) was used to examine idiopathic ERMs, identifying four distinct structural patterns distributed across different layers, extending from the ILM to the vitreous surface of the membranes. Specifically, they described: (a) thin, interwoven fibrous layers; (b) folded sheets of fibrous material; (c) thicker, more rigid layers composed of densely folded collagen fibrils; and (d) lacunar areas containing necrotic and/or inflammatory material [28].

Idiopathic ERMs contain predominantly glial cells with varying degrees of fibrosis, while secondary ERMs in proliferative vitreoretinopathy (PVR) or diabetic tractional detachments contain more diverse cellular populations and denser collagenous matrices [29].

Lamellar hole-associated epiretinal proliferation (LHEP) represents a distinct entity characterized by thick, yellow-white material at the macular surface composed primarily of fibroblasts, hyalocytes, and types I-III collagen without type IV collagen or fibronectin. This unique composition explains why LHEP does not stain with either trypan blue or brilliant blue G, creating characteristic “negative staining” patterns [30].

## Vital dyes: physicochemical properties and mechanisms

### Brilliant blue G

Brilliant blue G (BBG), also known as Coomassie Brilliant Blue G-250, is a triarylmethane dye with a molecular weight of 854 Da. It has been introduced as a surgical adjuvant for chromovitrectomy in 2006 [31, 32] and, this dye was reported to stain the ILM more than ERMs [33].

The molecular structure of BBG features a triarylmethane chromophore and sulfonic acid groups, which facilitate high water solubility and strong protein binding affinity. BBG binds preferentially to ILM components through combined electrostatic and hydrophobic interactions. Electrostatic binding occurs between negatively charged sulfonic groups and positively charged aminoacids (lysine, histidine, arginine), while hydrophobic forces facilitate interaction with aromatic residues (phenylalanine, tyrosine, tryptophan) [34]. This binding profile confers high ILM selectivity with limited penetration into underlying retinal layers.

Commercial BBG formulations for ophthalmic use are available at 0.025% concentration with physiological pH (7.0–7.6) and osmolality (301–400 mOsm/L) [35]. There are a number of commercially available preparations: ILM-Blue 0.025% (DORC, Zuidland, Netherlands); Brilliant Peel 0.025% (Fluoron, Ulm, Germany); ILM dye 0.025% (Meran, Istanbul, Turkey); and OcuBlue Plus 0.05% (Aurolab, Tamil Nadu, India). Different preclinical and clinical studies showed that a clinical concentration of 0.025% represents a balance between adequate staining and biocompatibility.

The retinal pigment epithelium (RPE) cell line ARPE-19 has commonly been used as an in vitro model to assess the safety profile of BBG. Overall, safety outcomes were considered acceptable at the dye concentration (0.25 mg/ml; 0.025%) and exposure times typically applied in clinical settings. However, several studies reported signs of cellular damage and decreased viability at higher concentrations (> 0.5 mg/ml) and with prolonged exposure (> 5 min) [36, 37].

Animal in vivo models showed a good safety profile with only transient changes observed in electrophysiological exams [38, 39].

Following encouraging preclinical results, multiple clinical studies evaluated the safety of Brilliant Blue G (BBG), often comparing it with Indocyanine Green (ICG), the previously preferred dye. Early retrospective and randomized studies in macular hole (MH) surgery found no significant differences in best-corrected visual acuity (BCVA), visual fields, or OCT parameters between BBG and ICG [40]. However, some evidence showed reduced photopic electroretinogram responses in ICG-treated patients [41]. Randomized and retrospective trials generally reported similar functional outcomes between BBG and ICG, though one database study found better BCVA and higher MH closure rates with BBG [42]. OCT analysis after BBG-assisted surgery revealed thinning in the temporal macular quadrant, possibly due to natural anatomical vulnerability or mechanical trauma during peeling rather than dye toxicity [43].

Comparisons with triamcinolone (TA) showed similar visual and anatomical results, although TA was associated with transient postoperative intraocular pressure elevation. BBG often produced better final BCVA than ICG and comparable outcomes to TA [44, 45].

Histological studies demonstrated that all dyes altered the ILM cleavage plane, but changes were more pronounced with ICG, while BBG caused minimal cellular debris. Numerous clinical series confirmed the general safety of 0.25 mg/ml BBG, with no BBG-related complications and stable retinal layer measurements [46, 47].

Rare adverse events, such as subretinal migration with RPE atrophy, were described. BBG has also shown good safety profiles in other vitreoretinal surgeries, including epiretinal membrane and myopic foveoschisis procedures, with no significant toxicity reported [48].

### Trypan blue

Trypan blue (TB) is a tetrasulfonated bis-azo dye with molecular weight 961 Da [49]. It is widely employed in medical histology due to its capacity to stain amyloid deposits, collagen fibers, and nonviable tissue [50]. In ophthalmology, it was initially introduced to facilitate capsulorhexis during cataract surgery and was later demonstrated to have a particular affinity for staining epiretinal membranes (ERM). Unlike BBG, TB demonstrates selective uptake by cells with compromised membrane integrity, explaining its affinity for ERMs containing dead or damaged glial cells rather than the acellular ILM [51].

The mechanism involves TB penetration through disrupted cell membranes with subsequent binding to intracellular proteins, producing characteristic blue coloration. Living cells with intact membranes exclude TB through active membrane transport mechanisms [52]. This property enables TB to distinguish viable from non-viable tissues, a principle also exploited in corneal endothelial assessment [53].

For vitreoretinal surgery, TB is available at concentrations from 0.09% to 0.18% [54].

Commercially available ophthalmic preparations for vitreoretinal surgery are the following: Monoblue NafX 0.15% (BVI, Waltham MA, USA), RetiBlue 0.15% (Aurolab, Tamil Nadu, India).

Higher concentrations (0.15–0.18%) are typically used for ERM and PVR membrane staining, while lower concentrations suffice for less cellular tissues. TB also binds to type IV collagen, enabling some ILM staining, though with less intensity than BBG [55].

Several studies have compared trypan blue (TB) and Indocyanine Green (ICG) in macular surgery. In a randomized controlled trial of 40 eyes, Beutel et al. found no significant postoperative BCVA difference between 0.5 mg/ml ICG and 1.5 mg/ml TB, although secondary analyses suggested greater visual improvement with TB [56].

Haritoglou et al. found no BCVA or visual field differences at 12 months between ERM surgery performed with or without 0.6 mg/ml TB, attributing minor macular changes to membrane removal rather than dye toxicity[57]. In an adaptive RCT of 78 MH eyes, Christensen et al. showed that ILM peeling improved closure rates, with similar BCVA gains between TB and ICG in successfully closed holes [58].

Ultrastructural analysis showed no difference in cleavage plane or toxicity with TB-assisted peeling [59], and no RNFL thinning was observed after TB use [60]. Multiple case series further supported the safety of TB in vitreoretinal surgery, with no significant toxicity reported across various indications, including ERM, MH, and proliferative vitreoretinopathy [61, 62].

### Indocyanine green

Indocyanine green (ICG) is an amphiphilic tricarbocyanine dye with molecular weight 775 Da, originally developed for hepatic function testing and angiography [63]. Its strong binding to ILM laminin and type IV collagen produces intense green staining that historically made it popular for ILM peeling.

However, significant toxicity concerns have emerged regarding ICG use. Multiple mechanisms contribute to potential retinal damage:

1. **Osmolarity-related toxicity**: ICG powder dissolved in water produces hypotonic solutions that can disrupt retinal architecture [64].
2. **Phototoxicity**: ICG absorption spectrum (600–900 nm) overlaps significantly with surgical light sources, generating reactive oxygen species through photodynamic reactions [65].
3. **Direct cellular toxicity**: ICG induces apoptosis in RPE cells through mitochondrial pathways with Bax upregulation and Bcl-2 downregulation [36].
4. **Iodide content**: Commercial ICG contains up to 5% sodium iodide, which may contribute to retinal toxicity [66].

Meta-analyses comparing ICG with alternative dyes have consistently demonstrated inferior visual outcomes and higher rates of RPE abnormalities following ICG-assisted ILM peeling [67]. Consequently, ICG use has declined substantially, with blue dyes now preferred for most indications.

### Triamcinolone acetonide

Triamcinolone acetonide (TA) is a water-insoluble synthetic corticosteroid that, while not a true vital dye, enables visualization through a distinct mechanism. Its insoluble white crystals adsorb onto collagen fibrils of the vitreous gel, forming visible aggregates that delineate otherwise transparent vitreous structures [68, 69]. TA is particularly valuable for confirming posterior vitreous detachment, identifying vitreous cortex remnants after apparent PVD, visualizing the vitreous base during peripheral vitrectomy, and detecting residual vitreous in diabetic traction cases.

Preserved TA formulations (4%) have been used off-label for decades, though concerns regarding benzyl alcohol preservative toxicity prompted the development of preservative-free formulations specifically registered for vitrectomy use [70]. These preservative-free preparations contain uniform crystal dispersions at 4% or 8% concentrations without preservatives.

Triesence is a preservative-free TA suspension approved by the FDA for intraocular use, including vitreous visualization during surgery. It is supplied as a 1 mL vial at 40 mg/mL, with a recommended intravitreal dose of 1–4 mg. Trivaris is another preservative-free preparation, provided as an 8 mg (80 mg/mL) single-use syringe for intravitreal injection.

Kenalog is used off-label for similar purposes but contains benzyl alcohol as a preservative. Many surgeons, therefore, prefer preservative-free compounded versions. The typical concentration is 40 mg/mL, and during vitrectomy TA is commonly diluted 1:4 with balanced salt solution before intraocular administration.

A critical consideration is that TA crystals demonstrate strong adherence to exposed neural tissue. After ILM peeling, TA should not be applied as crystals can deposit on denuded Müller cell endfeet, with case reports of persistent foveal deposits and delayed visual recovery [71].

### Comparative dye characteristics

Table 1.

**Table 1:** Comparative properties of vital dyes for chromovitrectomy

Property	Brilliant Blue G	Trypan Blue	Indocyanine Green	Triamcinolone Acetonide
Chemical class	Triarylmethane	Azo dye	Cyanine	Corticosteroid
Molecular weight (Da)	854	961	775	435
Clinical concentration	0.025%	0.09–0.18%	≤ 0.05%*	4%
Primary target	ILM	ERM, dead cells	ILM	Vitreous
Binding mechanism	Electrostatic + hydrophobic	Intracellular uptake	Laminin/collagen IV	Crystal adsorption
Regulatory status	Approved	Approved	Off-label	Off-label/Approved**
Phototoxicity risk	Low	Low	High	Minimal
Recommended exposure	< 60 s	< 60 s	< 30 s	Immediate removal

*ICG use declining due to safety concerns; **Preservative-free formulations approved in some jurisdictions

## Advanced dye formulations

### Heavy dyes with sinking agents

A significant advancement in chromovitrectomy has been the development of “heavy” dye formulations containing density-increasing agents that facilitate targeted delivery to the posterior pole [72]. These formulations, with specific gravity exceeding 1.02 g/mL, sink rapidly through balanced salt solution to settle on the macula without dispersing throughout the vitreous cavity.

Available sinking agents include polyethylene glycol (PEG), which provides membrane-stabilizing and antioxidant properties beyond density enhancement [73]; deuterated water (D₂O), which increases density by approximately 10% with minimal chemical interaction [74]; diglycerol, offering lower viscosity than PEG and improving injection characteristics [75]; and soluble lutein, which acts as both sinking agent and potential photoprotectant [76].

Heavy dye advantages include targeted macular delivery without peripheral retina exposure, reduced dilution factor compared to intravitreal injection, elimination of air-filled eye technique requirement, and shorter staining times due to concentrated tissue contact.

Commercial heavy dye formulations include 0.025% BBG with 4% PEG (e.g., ILM-Blue^®^), 0.15% TB combined with 0.025% BBG and 4% PEG (MembraneBlue-Dual^®^), and newer preparations containing DDG (a BBG derivative with methoxy substitution) combined with diglycerol (e.g. MonoBlue Dual View^®^) [73].

PEG use in ARPE-19 cell line showed a reduction in dye toxicity when compared to TB alone, BBG alone or a combination of them without PEG [77].

### BBG derivatives

Novel BBG derivatives have been engineered to enhance ILM selectivity and reduce retinal penetration. Three patented compounds, pure benzyl-BBG (PBB), DDG, and methyl-BBG (Blulife), incorporate structural modifications to the diphenylamine nitrogen that increase lipophilicity and hydrophobic protein interactions [34].

In silico molecular docking and in vitro binding studies demonstrate that PBB exhibits stronger van der Waals interactions with fibronectin hydrophobic residues compared to traditional BBG [78]. Ex vivo experiments confirm that PBB is sequestered by ILM with negligible diffusion into underlying retinal layers, potentially offering an improved safety profile [34].

DDG (containing methoxy instead of ethoxy groups) offers greater transparency than BBG during staining, improving posterior pole visualization while maintaining equivalent protein binding [75]. Clinical comparative data remain limited, though early experience suggests comparable efficacy with potentially enhanced visibility.

### Combined dye formulations

Combining BBG and TB leverages their complementary binding profiles, BBG for ILM and TB for cellular ERM components, to achieve simultaneous visualization of multiple tissue types [79]. These “dual” dyes enable surgeons to distinguish ILM from overlying ERM based on differential staining patterns: homogeneous blue indicates ILM predominantly; intense focal staining indicates ERM with cellular components; patchy staining suggests mixed ILM/ERM with variable cellular content; and absent staining indicates LHEP, vitreoschisis, or hypercellular tissue [72, 80].

Available combined formulations include 0.15% TB + 0.025% BBG + 4% PEG (MembraneBlue-Dual^®^), 0.18% TB + 0.03% Blulife (methyl-BBG) + thickening agent (TWIN^®^), and 0.09% TB + 0.025% DDG + 2.6% diglycerol (MonoBlue Dual View^®^).

Among these, the last one is the only one formed by a combination of TB with DDG, a new non-toxic dye used in substitution of BBG, and with diglycerol as osmotically benign agent to enhance density. The use of diglycerol, replacing PEG polymers, allows to decrease the concentration of TB in the staining solutions from 0.15% to 0.09% with maintaining the same staining levels [81].

Comparative studies suggest combined dyes provide equivalent or superior staining efficacy compared to single dyes without increased toxicity, as the component concentrations remain within established safety thresholds [82]. Awad et al. analyzed BBG and TB combined effect on ARPE-19 cells in vitro finding that BBG at concentrations of 0.025% protected against TB-induced damage at 5 min and 30 min incubation [83].

Of practical relevance, the heavier and more viscous combined formulations, particularly those containing PEG or other thickening agents, may require longer intraocular aspiration times for complete washout compared with monomeric BBG solutions.

### Lutein-based formulations

Lutein, a naturally occurring xanthophyll carotenoid, represents an innovative approach combining staining capability with potential photoprotection [84]. Its amphiphilic structure enables interaction with both hydrophobic collagen components and hydrophilic membrane interfaces through a physical mechanism distinct from chemical dye binding [85].

Proposed advantages of lutein-based dyes include blue light filtering properties (absorption peak 445 nm), antioxidant capacity reducing reactive oxygen species generation, different interaction mechanisms potentially reducing cellular toxicity, and possible neuroprotective effects on retinal tissue thanks to the antioxidant properties of lutein and zeaxanthin [86, 87].

The introduction of a lutein-based vitreous dye (LB-VD; Vitreo Lutein) offers a potentially safer alternative to TA-based formulations [88]. Lutein use in combination with TB or BBG dyes enables better visualization of the ERMs as well.

A randomized clinical trial comparing lutein-based dye (0.15% TB + 0.05% BBG + 2% lutein) against standard dual dye (0.15% TB + 0.025% BBG + 4% PEG) and ICG for ERM surgery demonstrated faster visual recovery and improved macular sensitivity at early follow-up in the lutein group, though differences equalized by 6 months [89]. Histopathological analysis revealed reduced glial fibrillary acidic protein expression in peeled ILM specimens from lutein-treated eyes, suggesting less Müller cell trauma [89].

## Clinical applications and surgical techniques

### Macular hole surgery

ILM peeling has become standard treatment for full-thickness macular holes (FTMH), with closure rates exceeding 90% in primary surgery [90]. BBG or BBG-containing formulations are preferred for ILM visualization given their selectivity and safety profile.

Technique considerations include dye delivery injected slowly over the macula with infusion temporarily reduced to minimize turbulence; staining time of 30–60 s typically sufficient, following manufacturer recommendations; dye removal via complete aspiration through flute needle or active cutter suction before peeling initiation; and illumination reduction with maintained distance from macula during dye contact.

Apparently weak ILM staining is often due to residual cortical vitreous masking the underlying basement membrane, and a gentle preliminary peeling attempt over the stained area may expose well-stained ILM beneath, often obviating the need for repeat dye instillation.

For large or chronic macular holes, inverted ILM flap techniques require adequate tissue visualization. Some surgeons perform initial dye application, partial peeling to create the flap, then gentle second application to confirm flap positioning, though restaining over exposed retina increases toxicity risk and should be minimized [91].

Meta-analyses comparing BBG-assisted versus ICG-assisted ILM peeling for FTMH demonstrate superior visual outcomes with BBG, including better final visual acuity and reduced scotoma incidence [92].

### Epiretinal membrane surgery

ERM surgery benefits from dyes with affinity for both cellular membrane components (TB) and underlying ILM (BBG). Combined dual dyes or sequential staining protocols enable surgeons to identify ERM extent and edges, distinguish ERM from ILM during peeling, confirm complete ILM removal after ERM peeling, and recognize LHEP by its characteristic non-staining pattern.

“Negative staining” technique: When using BBG alone, ERMs appear as unstained areas contrasting with surrounding blue-stained ILM [93]. This enables ERM identification without TB-related concerns, allowing initiation of peeling at the ERM-ILM junction and combined removal in suitable cases.

For recurrent or secondary ERMs, more intense staining with combined dyes may be necessary to delineate fibrotic tissue planes. PVR membranes in particular benefit from higher TB concentrations (0.15–0.18%) that enhance visualization of the often-subtle proliferative tissue [94].

### Retinal detachment surgery

Retinal detachment surgery presents unique chromovitrectomy challenges, requiring visualization of multiple tissue types including vitreous, proliferative membranes, and retinal breaks. A systematic approach to dye application can significantly improve surgical outcomes.

#### Vitreous visualization

TA remains the preferred agent for vitreous visualization given its unique ability to delineate gel structure. Key applications include:

##### PVD confirmation

TA injected over the optic disc creates visible turbulence patterns, smooth dispersal indicates complete PVD, while localized accumulation suggests residual attachment [95, 96].

##### Vitreous cortex remnant identification

After apparent PVD, TA can reveal persistent cortical vitreous that may serve as a scaffold for cellular proliferation in PVR [97, 98]. This is particularly valuable in retinal detachment surgery where complete vitreous removal correlates with reduced reproliferation risk.

##### Vitreous base visualization

For diabetic tractional detachments and giant retinal tears, TA-assisted identification of vitreous base extent enables more complete peripheral vitrectomy [99].

##### Technique

TA should be injected with moderate velocity to entrap crystals within vitreous architecture. Excess crystals are immediately aspirated. The staining effect diminishes with continued surgery as crystals are progressively removed, repeat application may be necessary for extended procedures.

#### PVR membrane identification

Proliferative vitreoretinopathy membranes pose particular visualization challenges due to their variable cellularity and often-subtle appearance. TB at higher concentrations (0.15–0.18%) effectively stains cellular components of PVR membranes, facilitating complete removal [94]. Combined BBG/TB formulations enable simultaneous visualization of both cellular PVR tissue and any associated ILM involvement.

During silicone oil removal, residual PVR membranes may be identified using TB injection under oil, allowing targeted membrane peeling before oil-BSS exchange [100]. This technique can identify subclinical reproliferation that might otherwise lead to redetachment.

#### Retinal break identification with trypan blue

An important and underutilized application of TB is identification of occult retinal breaks during retinal detachment surgery. This technique is particularly valuable when preoperative examination fails to identify causative break(s).

##### Technique

After subretinal fluid drainage and retinal flattening with perfluorocarbon liquid (PFCL), diluted TB (typically 1:1 with BSS) is injected over suspected break locations [101]. The dye flows through patent retinal breaks into the subretinal space, identifying their location by the visible dye stream. Alternatively, TB can be injected subretinally through a retinotomy, with dye emerging through breaks when PFCL is used to express it [102, 103].

Jackson et al. described the subretinal injection technique using a 41-gauge cannula, demonstrating successful break identification in cases where standard visualization had failed [104]. The technique carries theoretical risk of RPE toxicity from direct dye contact, though clinical reports have not demonstrated significant adverse effects when diluted preparations are used briefly.

This technique has proven particularly valuable in inferior retinal detachments with multiple potential breaks, highly myopic eyes with peripheral degeneration, redetachment cases following initially successful surgery, and pediatric retinal detachments where cooperation limits examination.

#### Sequential staining protocol for complex retinal detachment

For complex retinal detachments, particularly those with PVR or without identifiable breaks, a sequential staining approach optimizes visualization at each surgical stage:

1. Initial TA application: Triamcinolone acetonide is applied to confirm PVD status and identify residual vitreous cortex remnants. A thorough core and peripheral vitrectomy are then completed, followed by complete TA removal.
2. TB for membrane/break identification: Trypan blue (0.15%) is applied to delineate PVR membranes and/or occult retinal breaks.
3. BBG for ILM assessment (if indicated): In cases requiring ILM peeling for macular pathology or to reduce PVR recurrence risk, apply BBG after TB removal.

To maximize dye contrast at the tissue surface, an optional air/BSS exchange may be performed prior to TB application; staining under air minimizes dye dilution and enhances concentration at the membrane interface. Following a brief dwell time, a second BSS/air exchange is performed to displace excess unbound dye and establish a fluid interface suitable for subsequent steps. Perfluorocarbon liquid (PFCL) is then injected, serving a dual purpose: mechanical tamponade to stabilize and flatten the detached retina, and further enhancement of tissue staining intensity. Membrane peeling and retinal break treatment are subsequently performed under PFCL. (Video 1)

This systematic approach ensures each tissue type is adequately visualized while minimizing cumulative dye exposure through complete removal between applications.

### Macular protection strategies

Several techniques have been described to protect macular hole edges or foveal tissue from dye exposure or mechanical trauma:

#### PFCL bubble protection

A small PFCL bubble placed over the macular hole before dye injection prevents dye entry into the subretinal space and direct RPE contact [105, 106].

#### Viscoelastic protection

Cohesive viscoelastic (e.g., sodium hyaluronate) placed over the fovea may reduce dye concentration at the hole edge, though this can also impair visualization [107].

#### Autologous blood

For inverted flap techniques, autologous blood applied to the flap may promote adhesion while creating a barrier to dye penetration [108].

## Digital visualization and chromovitrectomy

The introduction of three-dimensional (3D) heads-up display (HUD) surgical systems, exemplified by the Ngenuity platform (Alcon) and ARTEVO 800 (Zeiss), represents a paradigm shift in vitreoretinal surgery visualization.^55^ These systems employ high-dynamic-range cameras capturing surgical field images that are processed and displayed on large 3D monitors, with surgeons viewing through passive polarized glasses.

### Reduced illumination requirements

perhaps the most significant advantage for chromovitrectomy is the ability to operate at substantially reduced endoillumination levels. Digital gain compensation enables adequate visualization at reportedly 10–50% of conventional light intensity, significantly decreasing phototoxicity risk [109, 110]. Given the recognized synergistic toxicity between vital dyes and light exposure, this reduction may substantially improve chromovitrectomy safety [111].

### Enhanced dye visualization

**Fig. 1:**
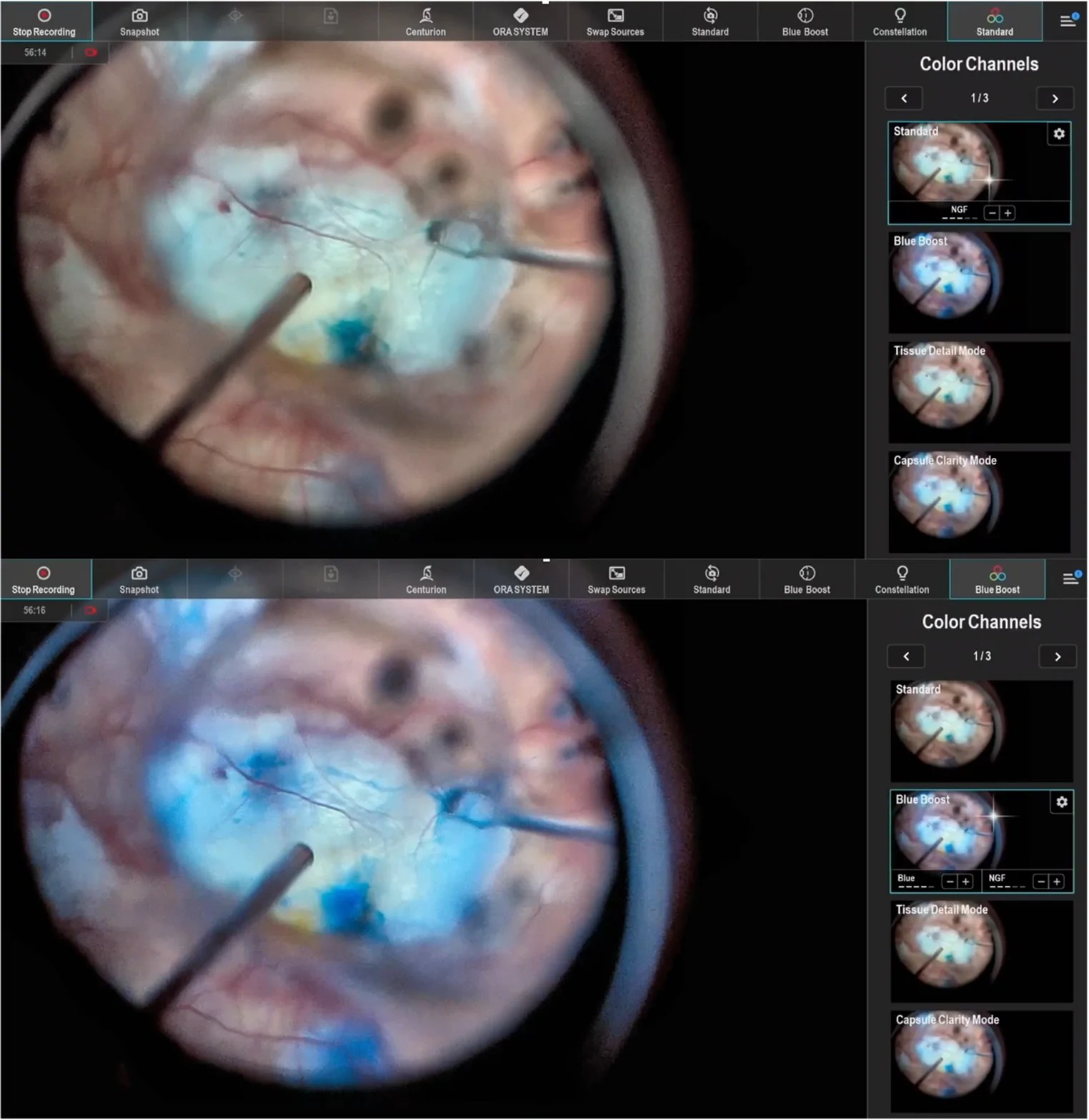
Digital enhancement of chromovitrectomy visualization. Intraoperative comparison of posterior segment staining using the NGENUITY^®^ 3D visualization system (Alcon Laboratories, Inc., Fort Worth, TX, USA). (Top) Standard digital visualization of Trypan Blue (0.15%). In the setting of pathological myopia and retinochoroidosis, the pale, atrophic fundus significantly reduces the contrast of the vital dye, making the ILM and the margins of the macular hole difficult to discern. (Bottom) Activation of the “Blue Boost” filter which significantly amplifies the saturation and luminance of the blue spectrum

digital processing amplifies subtle color differences between stained and unstained tissues, making dye visualization more apparent than through traditional optical microscopes [109]. Surgeons can adjust RGB channel balance intraoperatively, increasing blue channel gain improves BBG and TB visualization, while green channel adjustments may help identify residual ICG in revision cases [112]. Contrast enhancement algorithms can further amplify membrane edges, though excessive enhancement may introduce artifacts requiring optimization based on surgeon experience. (Fig. 1)

### Spectral optimization and photoprotection

Advanced ecosystems, such as the Alcon UNITY^®^, incorporate specialized digital modes like the Light Sentry™ yellow filter. This mode selectively filters high-energy, short-wavelength blue light, ensuring compliance with aphakic hazard standards. This is particularly critical during the air-interface phase of chromovitrectomy, where the change in refractive index often exacerbates light scattering and glare. By optimizing the spectral profile, the yellow filter enhances the contrast of stained preretinal tissues while mitigating the risk of light-induced macular injury during prolonged complex procedures.

### Educational value

The large display enables real-time observation by trainees and staff, improving educational efficiency and surgical team communication.

The interplay between digital enhancement and chromovitrectomy creates opportunities for reduced dye exposure. Lighter application may suffice when digital enhancement is available, potentially improving safety while maintaining visualization adequacy [113]. However, digital enhancement optimizes visualization of properly stained tissues rather than compensating for insufficient staining. Both Ngenuity and ARTEVO 800 offer proprietary enhancement features; specific capabilities vary by platform and software version, and surgeons should familiarize themselves with their system through manufacturer training.

## Safety considerations and phototoxicity

### Mechanisms of dye-related toxicity

Vital dye toxicity occurs through multiple pathways. Direct cytotoxicity results from chemical interaction with cellular components, causing dysfunction or death in a concentration and time-dependent manner [114]. Osmotic injury can occur when hypotonic dye solutions disrupt cellular architecture, a particular concern with ICG preparations reconstituted in water rather than balanced salt solution [115].

Phototoxicity represents a critical concern in chromovitrectomy. Light absorption by dyes generates reactive oxygen species through photodynamic reactions, damaging lipid membranes, proteins, and DNA [116] This mechanism underlies the importance of minimizing both dye concentration and illumination intensity, as the effects are synergistic rather than simply additive.

Additionally, sequential use of multiple surgical adjuncts, including dyes, PFCL, and silicone oil, may produce cumulative toxicity exceeding individual agent effects, though the precise interactions remain incompletely characterized [78, 117].

### Dye-specific toxicity profiles

#### Indocyanine green

carries the highest toxicity burden. Multiple mechanisms contribute: osmotic injury from hypotonic reconstitution, significant phototoxic potential due to absorption spectrum overlap with surgical illumination (600–900 nm), direct mitochondrial toxicity inducing RPE apoptosis, and possible iodide-related effects [36, 64–66].

In vitro studies with different kinds of retinal cells (ARPE-19 cells, glia cells, microglia, vascular endothelial cell and retinal neuronal cells) showed cytotoxicity towards specific retinal cell populations [41, 118, 119]. ICG intravitreous injections in rat eyes also showed severe alteration of retinal structure and partial atrophy of the RPE [120].

Clinical studies and meta-analyses consistently demonstrate inferior visual outcomes and higher rates of visual field defects following ICG-assisted surgery compared with alternative dyes [67]. RPE atrophy in the peeled area, sometimes progressive, has been reported even with “low-dose” ICG protocols. The occurrence of scattered unusual outer retinal and pigment epithelial changes on FA and ICGA has been noted in some cases, probably due to the enhanced phototoxicity associated with the use of ICG at a high concentration (0.5%) [121].

Consequently, ICG use has declined substantially worldwide, and many vitreoretinal surgeons have abandoned it entirely.

#### Brilliant blue G

demonstrates a favorable safety profile in both experimental and clinical studies. Its molecular structure limits retinal penetration, with dye largely sequestered at the ILM level [34]. Cytotoxicity studies show minimal RPE cell death at clinical concentrations (0.025%), with toxicity thresholds well above typical surgical exposure [122].

Animals in vivo studies showed a good safety profile, with no retinal thinning observed after intravitreal injection in rat eyes [123]. Ejstrup et al. also investigated the subretinal toxicity of BBG, finding no blood-retina barrier breakdown when performing FA [124].

However, BBG is not without risk, cases of presumed BBG-related toxicity have been reported, typically involving prolonged exposure, restaining over peeled ILM, or high illumination levels [125, 126]. The combination of BBG with light exposure can produce phototoxic effects, though significantly less than ICG [111].

#### Trypan blue

shows intermediate safety characteristics. At clinical concentrations (0.06–0.18%), TB demonstrates acceptable biocompatibility in cell culture models [114]. It also showed only temporary changes in rats’ electroretinograms, which completely recovered after 1 month from the intravitreal injections [39]. Its primary uptake by non-viable cells theoretically limits exposure of healthy retinal tissue. Clinical experience over two decades supports a good safety record, though direct subretinal exposure should be minimized given theoretical RPE toxicity concerns [127].

#### Triamcinolone acetonide

presents minimal direct chemical toxicity but unique mechanical considerations. Preservative-free formulations eliminate benzyl alcohol-related concerns [70]. The primary risk is crystal deposition on exposed neural tissue following ILM peeling, which can cause persistent foveal deposits and delayed visual recovery [71]. TA should therefore not be applied after ILM removal.

Subretinal injection of TA showed areas of absence and/or hyperpigmentation of the retinal pigment epithelium and damage to photoreceptors and the outer nuclear layer of the retina in rabbit eyes [128] and the injection of large quantities of TA in proximity to a macular hole should therefore be avoided.

#### Combined formulations

using BBG and TB at reduced individual concentrations appear to maintain the safety profiles of their components without additive toxicity, as each dye remains within established safe thresholds [73, 82].

### Clinical manifestations of toxicity

Reported cases of presumed chromovitrectomy-related toxicity share common features:

Symptoms include visual deterioration exceeding expected surgical outcomes, central or paracentral scotoma, and reduced contrast sensitivity. Patients may report a “smudge” or “gray spot” in central vision that persists beyond the expected recovery period.

Fundus findings include RPE mottling or stippling in the area of dye application, outer retina/RPE atrophy corresponding to the peeled zone, and occasionally progressive atrophy expansion over months following surgery [127].

OCT findings are often the most sensitive indicator, revealing outer retinal layer disruption, ellipsoid zone irregularity or loss, RPE thinning or atrophy, and foveal architectural changes [129]. These findings may be present even when fundus examination appears relatively normal.

Fundus autofluorescence demonstrates hypo- or hyperautofluorescent areas corresponding to RPE damage, often with sharply demarcated borders matching the area of dye exposure or ILM peeling [2].

Distinguishing dye toxicity from phototoxicity alone remains challenging, as clinical appearances overlap substantially. Most reported cases likely represent combined dye-light interaction rather than isolated dye toxicity [126]. This clinical reality reinforces the importance of minimizing both dye exposure and illumination intensity.

An additional aspect of dye-related retinal toxicity is the duration of intraretinal persistence in the postoperative period. Weinberger et al. demonstrated persistent intraretinal ICG fluorescence on infrared autofluorescence imaging up to six weeks after macular hole surgery, suggesting that the dye-tissue interaction, and any associated toxic effect, may extend well beyond the intraoperative phase [130].

## Future directions

### Novel dye development

Ongoing research explores natural-product-based dyes with improved biocompatibility. Anthocyanins derived from açaí fruit (Euterpe oleracea) have demonstrated ILM staining capability in experimental models with favorable toxicity profiles [131]. Other botanical compounds under investigation may offer combined staining and cytoprotective properties.

Nanotechnology approaches, including PLGA nanoparticle-encapsulated dyes, may provide enhanced stability, controlled release, and reduced toxicity compared to conventional formulations [132]. These delivery systems could enable longer staining duration without increased tissue exposure to free dye molecules.

### Artificial intelligence integration

AI-enhanced surgical systems may eventually provide automated tissue recognition reducing reliance on dye-based contrast, real-time dye concentration estimation from spectral analysis, cumulative light exposure monitoring with safety alerts, and predictive modeling of toxicity risk based on surgical parameters.

### Dye-free visualization

Advances in optical technologies raise the possibility of eventual dye-free chromovitrectomy alternatives, for example hyperspectral imaging exploiting native tissue optical properties and enhanced iOCT with en-face membrane visualization.

Intraoperative OCT (iOCT) has emerged as a complementary visualization modality to vital dyes. Microscope-integrated systems (RESCAN 700, EnFocus, Haag-Streit iOCT) provide real-time cross-sectional imaging of the vitreoretinal interface, enabling direct confirmation of ILM and ERM peeling completeness and identification of residual membranes. In the DISCOVER study, iOCT revealed unsuspected residual membrane in approximately 20% of peeling cases considered complete on microscopic view, and conversely confirmed complete removal in 40% of cases the surgeon believed still required peeling, thereby reducing unnecessary maneuvers [133]. Feasibility of stain-free, iOCT-guided ERM peeling has been demonstrated in selected cases, although vital dyes remain necessary for more complex peelings and for reliable ILM identification [134, 135].

Another option was proposed by Carlà et al. who integrated preoperative en-face OCT scans into the Alcon Ngenuity 3D system via a smartphone to guide epiretinal membrane (ERM) peeling. By projecting customized en-face slabs as a picture-in-picture display, they identified optimal cleavage points, achieving 100% successful peeling initiation at the first grasp without primary vital dye usage [136].

However, these technologies remain developmental, and vital dyes will likely remain essential surgical tools for the foreseeable future.

## Conclusions

Chromovitrectomy has evolved from empirical dye application to sophisticated, evidence-based practice integrating understanding of dye-tissue interactions, formulation optimization, digital visualization enhancement, and phototoxicity mitigation. Current evidence supports BBG and BBG-containing combined formulations as preferred choices for most vitreoretinal applications, with TB providing complementary ERM staining and TA remaining valuable for vitreous visualization.

The integration of 3D HUD surgical systems offers particular promise for improving chromovitrectomy safety through reduced illumination requirements while maintaining or enhancing visualization quality. Surgeons should recognize phototoxicity risks and employ evidence-based strategies to minimize dye exposure while achieving adequate tissue visualization.

Specialized techniques including TB-assisted retinal break identification, sequential staining protocols, and macular protection strategies expand the role of chromovitrectomy for complex cases. As novel dyes and visualization technologies emerge, the fundamental objective remains unchanged: enabling precise tissue identification and manipulation while preserving the neural retina from iatrogenic harm.

## Methods of literature search

A literature search was performed utilizing PubMed/MEDLINE, Embase, and the Cochrane Library databases for articles published between January 2005 and January 2026. The following search terms were employed: (“vital dyes” OR “brilliant blue G” OR “trypan blue” OR “indocyanine green” OR “chromovitrectomy”) AND (“membrane peeling” OR “ILM peeling” OR “macular hole surgery” OR “chromovitrectomy”). Further studies were located by manually searching the reference lists of pertinent articles. Foundational papers on anatomy, biochemistry, and early surgical techniques were included to establish context for the study. Inclusion criteria required peer-reviewed publications reporting original clinical or preclinical data regarding the application of vital dyes in vitreoretinal surgery. Case reports, case series, comparative studies, clinical trials, systematic reviews, meta-analyses, and preclinical investigations were deemed eligible. Review articles lacking original data were excluded; however, they were assessed for pertinent citations. A total of 425 potentially relevant articles were identified and subsequently screened based on their titles and abstracts. Following the initial screening, 133 articles satisfied the inclusion criteria and were incorporated into this review.

## Supplementary Information

Below is the link to the electronic supplementary material.

Supplementary Material 1 Sequential Staining and PFCL-Assisted Management of Complex Retinal Detachment.This video demonstrates a specialized staining protocol in a patient with a total retinal detachment complicated by proliferative vitreoretinopathy (PVR). During the air-interface phase, the UNITY® Light Sentry™ yellow filter (Alcon Laboratories, Inc., Fort Worth, TX, USA) is activated. The surgical sequence begins with an initial air-fluid exchange to create a dry environment for the application of Trypan Blue (TB, 0.15%). Staining "under air" is utilized to minimize dye dilution and maximize the concentration of the agent at the membrane interface. Following a brief dwell time, a bubble of perfluorocarbon liquid (PFCL) is injected into the eye. The PFCL serves a dual role: it provides mechanical tamponade to stabilize the mobile, detached retina and further enhances the staining intensity of the target tissues. The video concludes by showing the precise peeling of PVR membranes entirely under the PFCL bubble, ensuring maximum control and visibility in a highly complex surgical environment (MP4 55.6 MB)

## Data Availability

No datasets were generated or analysed during the current study.
